# Ultrastructural Features of Human Liver Specimens from Patients Who Died of Dengue Hemorrhagic Fever

**DOI:** 10.3390/tropicalmed4020063

**Published:** 2019-04-12

**Authors:** Min Min Win, Komgrid Charngkaew, Nuntaya Punyadee, Khin Saw Aye, Ne Win, Urai Chaisri, Nusara Chomanee, Panisadee Avirutnan, Sutee Yoksan, Prida Malasit

**Affiliations:** 1 Department of Tropical Pathology, Faculty of Tropical Medicine, Mahidol University, Bangkok 10400, Thailand; minwin28@gmail.com (M.M.W.); urai.cha@mahidol.ac.th (U.C.); 2 Department of Medical Research, Ministry of Health and Sports, Yangon 11191, Myanmar; ksadmr@gmail.com; 3 Department of Pathology, Pathology Research Division, Faculty of Medicine Siriraj Hospital, Mahidol University, Bangkok 10700, Thailand; k.charngkaew@gmail.com (K.C.); nchomanee@gmail.com (N.C.); 4 Division of Dengue Hemorrhagic Fever Research, Department of Research and Development, Faculty of Medicine Siriraj Hospital, Mahidol University, Bangkok 10700, Thailand; nuntayap@yahoo.com (N.P.); panisadee.avi@mahidol.ac.th (P.A.); 5 National Health Laboratory, Yangon 11181, Myanmar; drnewin07@gmail.com; 6 Center for Vaccine Development, Mahidol University, Nakhon Pathom 73170, Thailand; sutee.yok@mahidol.ac.th; 7 Medical Biotechnology Unit, National Center for Genetic Engineering and Biotechnology, National Science and Technology Development Agency, Bangkok 10400, Thailand

**Keywords:** dengue virus, dengue hemorrhagic fever, ultrastructure, electron microscope, suckling mouse brain, liver, autopsy

## Abstract

Recent advances in electron microscopy and tomography have revealed distinct virus-induced endoplasmic reticulum (ER) structures unique for dengue virus (DV) and other flaviviruses in cell culture models, including hepatocytes. These altered ultrastructures serve as sites for viral replication. In this study, we used transmission electron microscopy to investigate whether such structures were present in the liver of fatal dengue hemorrhagic fever (DHF) autopsy cases. In parallel, electron microscopic examination of suckling mouse brains experimentally infected with DV was performed as an in vivo model of acute DV infection. Typical features of ER changes containing abundance of replicative virions were observed in neurons and microglia of DV-infected suckling mouse brains (SMB). This indicated that the in vivo DV infection could induce similar viral replication structures as previously described in the in vitro DV-infected cell model. Nevertheless, liver tissues from autopsy of patients who died of DHF showed scant changes of ER membrane structures and rare particles of virions in hepatocytes, despite overwhelming evidence for the presence of viral antigens and RNA–indicating active virus replication. Instead hepatocytes contained an abundance of steatotic vesicles and structural damages. This lack of structural changes indicative of virus replication in human hepatocytes is discussed.

## 1. Introduction

Dengue viruses (DV) are mosquito-borne single-stranded RNA viruses within the family *Flaviviridae*. DV infections have become one of the most common diseases causing morbidity and mortality affecting millions of individuals worldwide [1]. The viruses, upon entering the cells, shed their coats and release their positive stranded RNA into the cytoplasm and begin translation into proteins. The process induces unique changes of the endoplasmic reticulum (ER) facilitating the replication of the RNA and packaging and exporting new virions. Recent applications of advanced electron microscopic technologies in the in vitro studies of flavivirus-infected cells have defined a set of distinct virus-induced endoplasmic membrane structures specific for DV and closely associated viruses including West Nile and Tick Borne Encephalitis [2]. The advanced studies have also described the functional aspects and interactions between the viruses and host molecular systems (reviewed in [3]). The structural features include: membrane invaginations into the ER lumen forming intra luminal vesicles (Ve) of ~90 nm which accumulate as vesicle packets (VP) (some vesicles maintain pore-like connections to the cytoplasm) and collections of convoluted membrane structures (CM) and membrane tubules (T) [4,5]. Virions are found within the ER membrane cisternae. These induced structures function as viral replicating factories where RNA translation, replication and packaging of the viral genome into virions take place [3]. Each new structure provides a particular function: Ve serves as a site for the “viral replicating complex” and a protected environment against host innate immune reactions to newly synthesized double stranded RNA [5,6], while CM is a site for RNA translation and viral protein processing [5,7]. The pattern of these cytoplasmic changes is found to be unique amongst closely related viruses within the same genera infecting mammalian and insect cells in vitro [5,6]. However, ultrastructural studies have not been carried out using in vivo infection models, where cell functions are under the influence of host physiological mechanisms. 

Our group has previously reported an autopsy study of 13 patients who died of severe dengue hemorrhagic fever (DHF) [8]. Using light microscopic and immunohistochemistry techniques, we have identified three major organs, i.e., liver, spleen and lymph nodes, that had significant levels of DV- RNA and – antigens, as well as histopathological changes. To further investigate ultrastructural changes in the human liver tissues, the present study used the same set of samples for transmission electron microscopy and searched for histopathology and modification of ER membrane structures, which are indicative of active viral replication, in the autopsy specimens. In addition, electron microscopic (EM) studies of brain tissues from suckling mice intra-cerebrally infected with DV were carried out in parallel to define the in vivo cytoplasmic structural changes during acute DV infection. The results showed scant features of endoplasmic changes and no obvious evidence of virion production in hepatocytes from DHF patients, but instead displayed abundant steatotic hepatic cell damages. Suckling mouse brains (SMB) infected with DV, on the other hand, showed typical features of ER changes in neurons and microglia similar to the observation in the in vitro hepatocyte infection model [2]. Some unique features of ER changes were also identified.

## 2. Materials and Methods

### 2.1. Liver Tissues from Patients Who Died of Severe Dengue Hemorrhagic Fever

Liver tissues were obtained from the autopsy project as reported by Aye et al. [8]. The study was conducted at the Yangon Children Hospital in 2005–2006, and was approved by the Siriraj Ethics Committee, Mahidol University (263/2551 [EC1]), the Ethics Review Committee, Department of Medical Research, Ministry of Health and Sports, Myanmar (24/Ethics 2003) and the International Vaccine Institute, Korea (institutional review board no. IRB-2005-6). Autopsies were conducted within 24 h after death to minimize autolysis and postmortem artifacts. Subjects were placed in the mortuary cold room of the Yangon Children Hospital (YCH), the internal temperature of which was kept at 4 °C to 8 °C constant.

### 2.2. DV Infection of Suckling Mice

Intra-cerebral inoculations of DV into 1-to-2-day-old suckling Swiss albino mice (purchased from the National Laboratory Animal Center of Mahidol University) were carried out using the Standard Operating Procedure of the Center for Vaccine Development that was approved by the Animal Experimental Committee of Mahidol University. Eight mice were inoculated with DV, two per each serotype and two mice were injected with equal volume of 50% fetal bovine serum/phosphate-buffered saline (FBS/PBS) and used as controls. 20 µL of DV suspension in 50% FBS/PBS containing 100 plaque forming unit [pfu]/mouse) was used for each mouse. Four serotypes were used: DV-1 Hawaii 199, DV-2 New Guinea C (NGC), DV-3 H-87, and DV-4 H-241. Following inoculation, the mice were observed twice daily for the appearance of signs of paralysis; if positive, the mice were sacrificed and tissues were collected and processed. Confirmation of the DV infection was retrospectively carried out by immunohistochemistry on the processed tissues using previously reported reagents and technology [8].

### 2.3. Specimen Processing for Electron Microscopy

#### Suckling Mouse Brains and Liver Tissues

The middle parts of the mouse cerebral hemispheres, about 1–2 mm proximal to the inoculation site, were removed and fixed in 2.5% glutaraldehyde in 0.1 M sodium cacodylate buffer at 4 °C overnight, and post-fixed in 1% osmium tetroxide in Millonig buffer for 1 h at 4 °C, and later dehydrated in a graded ethanol series and transferred to a 50:50 mixture of ethanol:propylene oxide and finally to 100% propylene oxide before being infiltrated with epoxy resin (Ted Pella Inc., Redding, CA) and polymerized at 60 °C for 48 h. Semi thin sections were cut, stained with Toluidine blue for light microscopic examination. Ultra-thin sections were cut with a Leica Ultracut UC7 microtome with a diamond knife, mounted on grids and stained with 2% uranyl acetate and lead citrate solution. The samples were examined using an FEI Tecnai™ T20 transmission electron microscope operated at 200 kV.

Parts of the cerebral hemispheres were fixed in 10% neutral-buffered formalin for 24 to 48 h and embedded into paraffin blocks for light microscopic examination and immunohistochemistry identifying the virus infected cells by serotype-specific monoclonal antibodies [8]. 

Liver tissues were taken from the right and left lobes of the liver. Small pieces of 1–2 mm cubes were diced, fixed and passed through similar processes as used in the SMB for EM study.

## 3. Results

### 3.1. Infection of the Liver

Table 1 provides overview of the demographic, clinical and laboratory processes and detailed data can be found in the publication [8]. Only specimens that yielded information needed for ultrastructural interpretations were selected (from 7 of 13 patients). All were children aged between 3 to 9 years of age who died 4 to 11 days after the first onset of fever. All had hemorrhagic manifestations, hepatomegaly, evidence of leakage and/or shock, ascites and pleural effusion; and were classified as dengue hemorrhagic fever or dengue shock syndrome (DHF/DSS) based on WHO 1997 classification [9]. Table 1 gives an overview of light microscopic, immunohistochemistry and viral information of the liver study taken from [8]: it shows extensive degree of hepatic necrosis, steatosis, Kupffer cell hyperplasia and hemophagocytosis. Dengue-specific RNA was found in the liver tissues, and immunohistochemistry showed the presence of dengue structural and non-structural proteins (envelope, NS1 and NS3) deposited within the cytoplasm of the hepatocytes. These previous findings suggest that hepatocytes in the liver are likely the site for DV replication in a susceptible human host.

**Figure 1:**
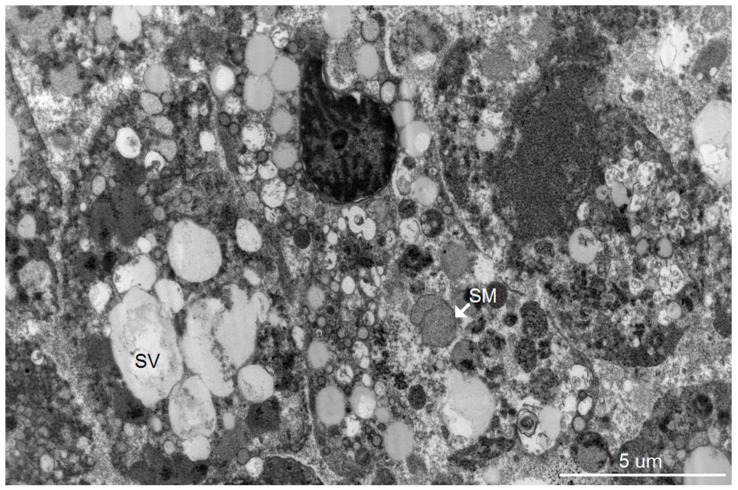
Electron micrograph depicting three highly damaged hepatocytes with differing degrees of nuclear changes from pyknotic to karyolitic. Cytoplasm of all the cells contains steatotic microvesicles (SV) and swollen mitochondria (SM). The right cell is disrupted with scattered cytoplasm.

**Figure 2:**
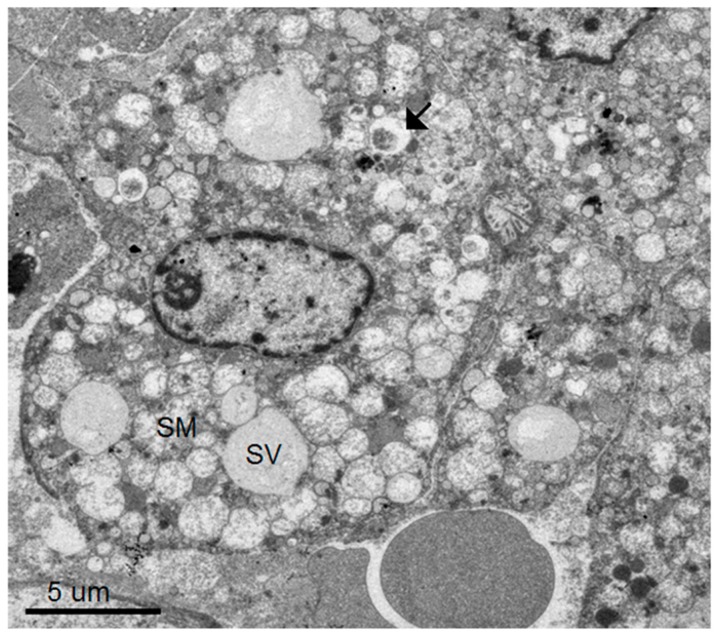
A hepatocyte with relatively intact nucleus and cytoplasmic membrane bordering a sinusoid with circulating red cells. Its cytoplasm contains steatotic vesicles (SV) alongside numerous swollen mitochondria (SM). Few lysosomes are identified (arrow).

**Figure 3:**
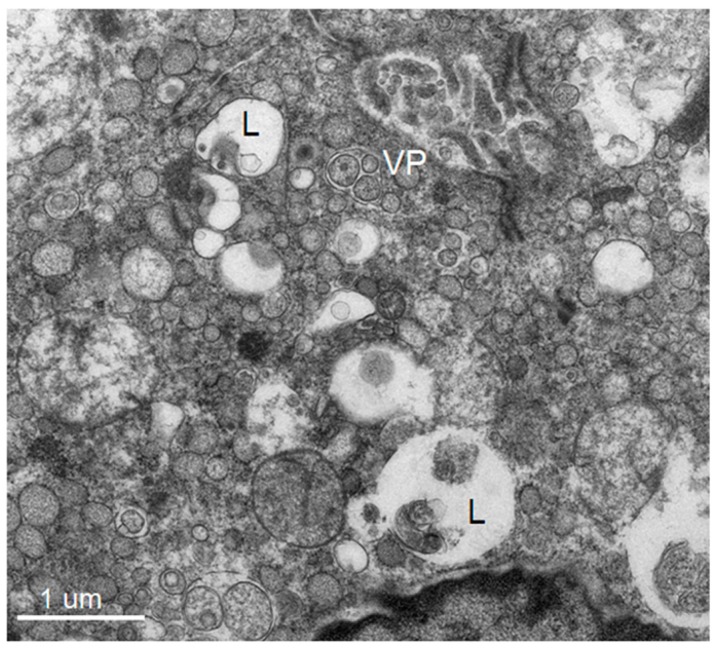
Perinuclear region showing an abundance of microvesicles of varying sizes interspersed with lysosomes containing round fragments of organelles (L). Few areas show features similar to the vesicular packets (VP), which are small vesicles residing within the enlarged ER. The vesicles are of varying sizes and not arranged in an orderly fashion as in classical vesicular packets.

**Figure 4:**
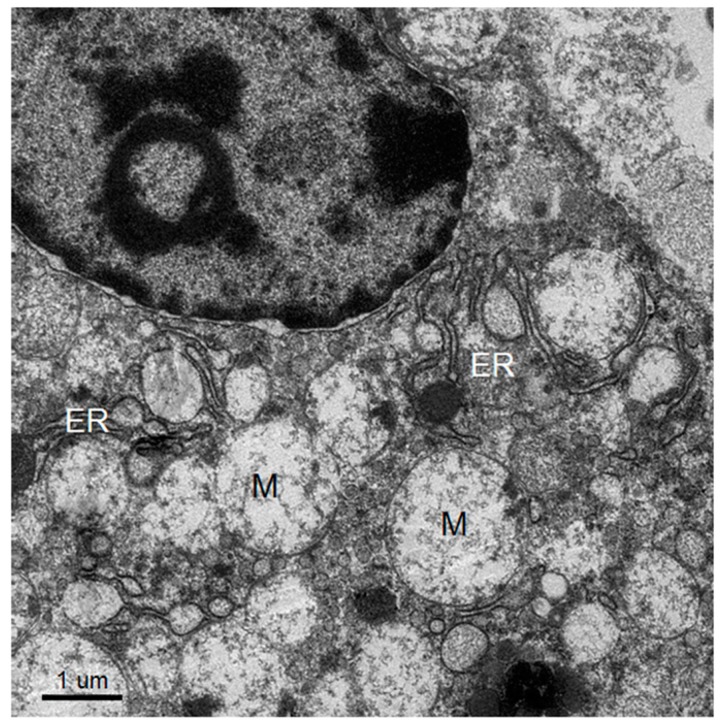
Perinuclear region of a relatively intact hepatocyte showing arrays of ER and Golgi apparatus, some with inflated cisternae squeezed between ballooning mitochondria (M). Neither vesicular packets, nor structures similar to CM, were found.

Dominant ultrastructural features of the hepatocytes from the seven patients showed a similar pattern of ultrastructural changes but with a variable degree of hepatocyte damage as follows: abundant lysosomes with varied content and sizes, vacuolization, steatosis and damaged and swollen mitochondria with distorted or damaged cisternae. Some enlarged and active phagocytic Kupffer cells were occasionally found. The dominant pathology of the hepatocytes was steatosis that was consistently observed (Figure 1, Figure 2, Figure 3 and Figure 4). Failure of electron microscopy to find any areas with significant immune cell infiltration was noted and in line with our previous results demonstrated by immunohistochemistry [8]. It was rare to find good intact hepatocytes with normal cytoplasm. Hepatocyte nuclei showed wide ranges of changes from normal to pyknotic nuclei. The ER and Golgi system were prominent in some cells. Dilated cisternae and/or vesicle formations were found, but were not as prominent as those described in the suckling mouse model (Figure 3). Typical VPs were found only in some areas and double membrane vesicles could occasionally be identified, particularly in areas where small variable vesicles were abundantly accumulated (Figure 3). Arrays of virions within vesicles could not be identified, and dubious solitary virions could occasionally be spotted, but were difficult to verify on its morphology alone (Figure 4). Attempts to find cytoplasmic areas containing aggregates of convoluted membranes failed. Despite ample evidence of hepatocyte damage, no viral induced replicating structure has been found in the liver tissues.

### 3.2. Infection of the Suckling Mouse Brains

**Figure 5:**
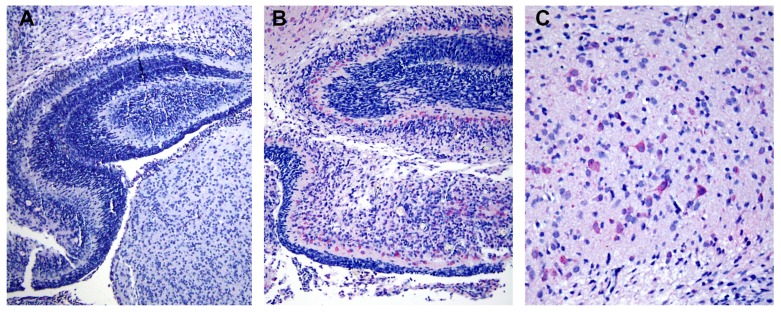
Localization of envelope (E) protein in cerebral cortex of dengue virus (DV) serotype 2 infected mice. (**A**) Low magnification (10 ×) of mouse brain tissue from control group showed negative staining. (**B**) Positive E staining (pink) in cytoplasm of neuronal cells was revealed by anti-E specific monoclonal antibody (clone 4G2) and alkaline phosphatase secondary antibody at low magnification (10 ×). (**C**) A high magnification (20 ×) of **B** showed distribution of E protein in the majority of neuronal cells in the cerebral cortex region.

**Figure 6:**
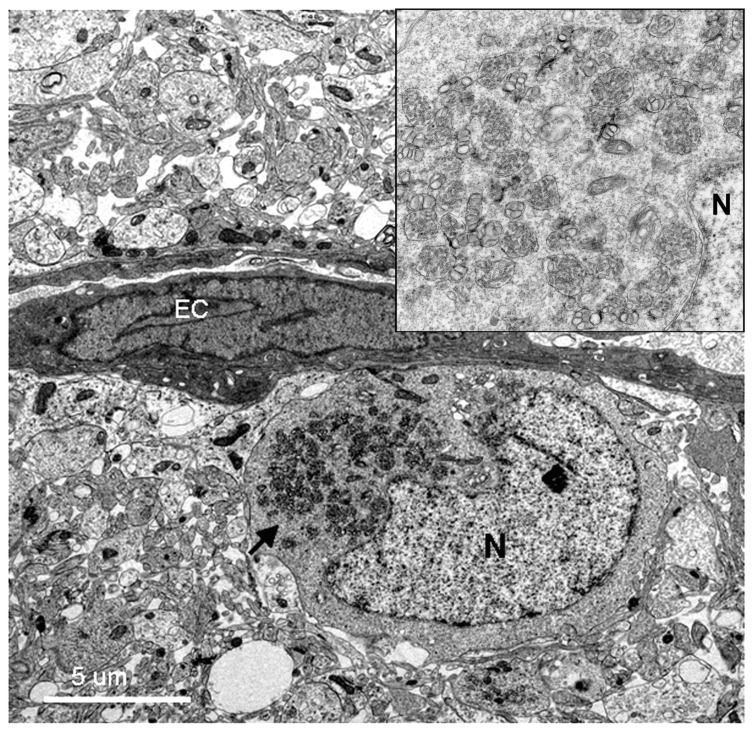
Low resolution electron micrograph of a DV-infected neuron near an endothelial cell lining capillary (EC). The perinuclear region of the cytoplasm of the neuron is filled with packets of virus-induced membranes (arrow pointing to high resolution inset at upper right corner). The packets are circumscribed by membranes. The nucleus and nucleolus (N) appear normal with intact nuclear membranes.

**Figure 7:**
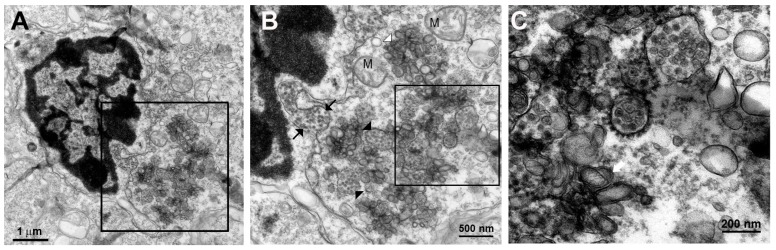
(**A**) Low power of an infected microglial cell showing typical irregular contour-shaped cell with dark nucleus with distinct peripheral heterochromatin condensation. Cytoplasmic area along the right of the nucleus shows dilated ER and collections of vesicles (inset). (**B**) Higher power of the perinuclear region showed a dilated ER (between black arrows) that courses along the nucleus, filled with dengue virions. The cytoplasm contains virus filled vesicles (black arrow heads) mixed with aggregates of double-membrane bodies (white arrowhead) and a group of swollen mitochondria (M) flanking the vesicles. (**C**) High power view of B inset (area within the rectangle) shows ribosome-studded ER containing dense core virions with a diameter of 40–50 nm. The core density and sizes of some of the intravesicular virions vary. Vesicular packets with round and oblong/tubular vesicles were observed (white arrow).

**Figure 8:**
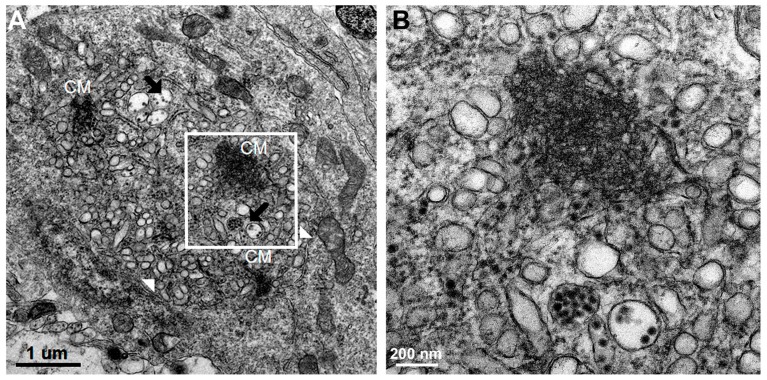
(**A**) Perinuclear region of an infected neuron depicts numbers of organelles, consisting of three groups of dense CMs, membrane bound vesicles containing arrays of densely-cored virions 40–50 nm in diameter (black arrows) and vesicles of different sizes and shapes (white arrow heads). (**B**) High power view of A inset (area within the rectangle) shows detail architecture of a CM which contains densely packed membrane aggregates.

All DV-infected SMB gave positive immunohistochemistry results with serotype-specific monoclonal antibodies [8], whereas controls were all negative for viral antigens (Figure 5). Neurons and microglia consistently showed signs of infection but no differences in pathological features were found amongst different serotypes. Neuronal, not endothelial cells showed packets of virus-induced membranes in perinuclear region of the cytoplasm (Figure 6). Alteration of the ER was the most conspicuous cytoplasmic change. The ER became more prominent, numerous and distended into cisternae containing closely packed vesicles of different shapes and contents. Some enlarged envelope-containing virions could also be identified (Figure 7A,B). Virus-induced ER membrane structures aggregated around the perinuclear region. The structures consisting of double membrane vesicles, densely packed convoluted membrane aggregates (CM), closely associated with dilated ER containing multiple vesicles or vesicular packets (VP), and round vesicles containing clusters of virions were found in the DV-infected mouse brain (Figure 8; CM and VP were described in Reference [3]). These features have recently been defined by a series of in vitro DV-infected cell culture studies that culminated in a list of hallmark features unique for DV and its closely associated flaviviruses [3]. Our results demonstrated that these features also occur in an in vivo infection of a mouse brain.

**Figure 9:**
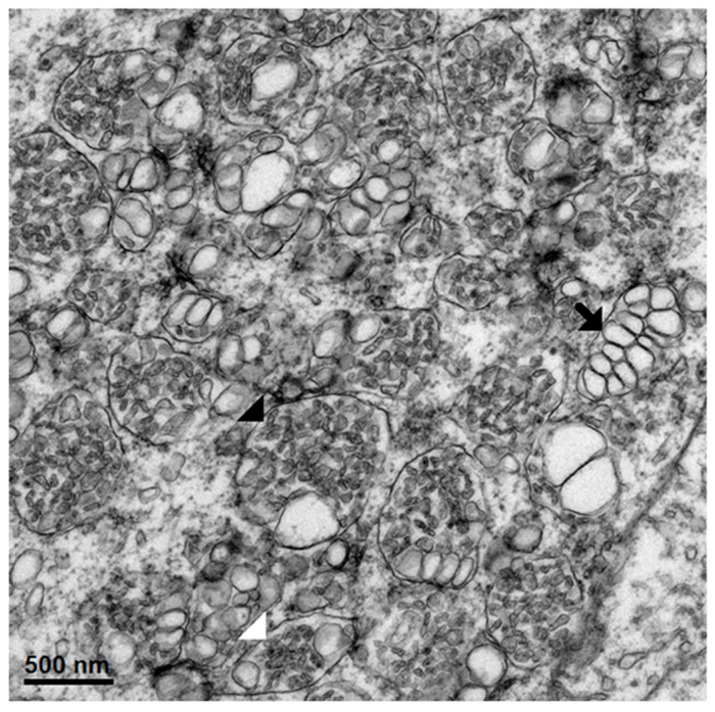
A cytoplasmic area containing a plethora of vesicular packets with different sizes and shapes of content: virion-like pellets, rounded vesicles and some with vesicular tubules of different length. Many large vesicles contain large circular vesicles devoid of content (black arrows), some contain deformed particles (black arrow head), some with a dense core resembling virions (white arrow head).

Additionally, we observed some unique features in DV-infected mouse brain as shown in Figure 9. The enlarged ER cisternae were filled with varying sizes and shapes of “pellets”, i.e., round, oblong, short tubules resembling structures of deformed virions. All were mixed with other well-formed empty vesicles of different sizes and packaged within intact membranes. These structures were different from the CM, which is an aggregate of membranes within the cytoplasm without any limiting membrane. The formation of intra-cisterna vesicles was caused by the synthesis of non-structural viral proteins at the rough ER membrane which induces invagination of the membrane forming vesicles [5]. Therefore, most of the contents within the cisternae have been induced by viral proteins. We interpreted the findings of excess variety of contents within the cisternae as a result of hypersensitivity of the membrane system of the host to the virus induction in in vivo infection. This might be relevant to DV infection in human hosts.

**Figure 10:**
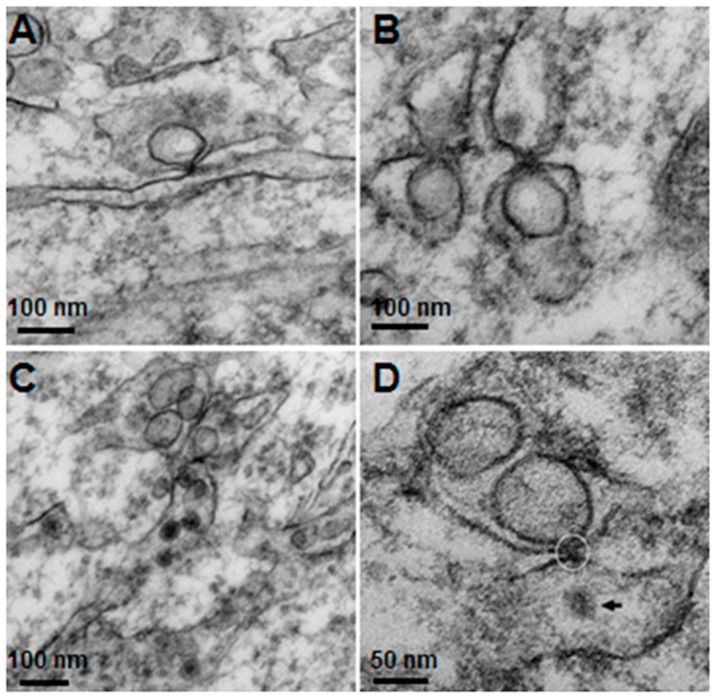
Invaginated vesicles and the opposite endoplasmic reticulum (ER), the points where the vesicle touches the apposing membrane are seen as electron dense knobs. (**A**) shows one vesicle touching an ER ready to deliver its content to the neighboring ER. (**B**) shows two pairs of apposing vesicles, the lower vesicles deliver the contents to the apposing ER via the touching knobs; the right pair shows a newly budding virus in the upper ER. (**C**) shows a vesicle touching the lower ER containing few virions. (**D**) shows the point of attachment (circle) between a vesicle and ER with a presumed new virion (black arrow) in the lower chamber.

Welsch et al. [5] demonstrated the viral budding process by showing the vesicle pore connecting to the cytoplasm to release the newly synthesized viral RNA from within to pass through the cytoplasm and bud to form new virions via the ER membrane opposing the pore. In this study, we also found these presumed budding sites in DV-infected mouse brain (Figure 10). Although we were not able to demonstrate the pores, we did observe sites where one vesicle situated within its ER sac, “fused” tangentially with its membrane that in turn touched the opposing ER membrane containing newly formed virions. These sites could be identified by careful searching, particularly in areas where there were active vesicular packets and active presence of virions and CM.

## 4. Discussion

Our study is the most comprehensive ultrastructural study of the liver from patients who died of severe DHF. Specimens designed for EM study from seven patients were studied. The study was a follow up from the histopathology study reported in 2014 by Aye et al., using materials systematically and properly collected from a group of children who died of DHF/DSS [8]. In the previous study, the liver was one of the key organs found to be infected. DV antigens (both structural and non-structural) were identified in hepatocytes by immunohistochemistry methods; DV-specific RNAs were also detected and quantified from liver tissue extracts. The degree of damage to the liver has been systematically studied and quantified. Two surprising findings in this current study are the fact that there were very few cytoplasmic structural changes compatible with efficient virus reproduction as those found in the suckling mouse brain, despite overwhelming evidence of the presence of viral antigens and RNAs in the tissues at the time of autopsy [8]. In addition, the pattern of responses by patients’ liver cells is completely different from the manner in which the hepatocyte cell lines, i.e., HepG2 or Huh-7 responded in vitro to the viruses [5]. 

Using transmission electron microscopy, we confirmed all of the hallmarks of cytoplasmic membrane changes induced by DV infection of mouse neurons and microglial cells in the in vivo suckling mouse brain infection model. These features had recently been defined by electron tomography and modern cryo-preservative fixation applied to infections in mammalian and insect-cell cultures. The structures, when combined with associated functional studies as carried out by immuno-electron microscopic and molecular studies, gave a complete picture of the molecular mechanisms of how the replication and formation of new virions occurred (reviewed in [3]). We also demonstrated some unique features that were found in the in vivo infections. First, there was a higher degree of membrane proliferative changes, with different shapes of intra-membranous vesicles ranging from rounded, oblong and tubular shapes, arranged in packets and distributed around the perinuclear area. Second, apart from newly formed virions with a size of 50 nm sizes and electron-dense nuclei, we also frequently found deformed and defective “virion-like vesicles” packed inside large vesicles. The latter features have not been previously described in in vitro infections. It was not known whether they were the result of excess but defective virus budding or if they were simply due to excessive induction of vesicles.

The knowledge of virus budding processes gained from the recent tomographic studies [5] allowed us to search for similar structures and sites where the new virions are formed by budding in infected mouse brain. We could identify the particular areas where two opposing ER meet, forming an electron dense “spot”. On one side there is usually an intra-ER vesicle touching the spot of the opposing bare membrane of the neighboring ER (Figure 5). According to the three-dimensional (3D) structure and tomographic studies [5,6], the site is the spot where the mouth of an invaginated vesicle containing the virus replicating complex meets the opposing ER membrane, delivering the positive RNA to the membrane and inducing the budding and formation of new virions in the ER cisternae. These spots were frequently found if carefully searched for newly formed virions that were usually found nearby.

Interestingly, despite the fact that there was extensive ER proliferation and Golgi structural changes facilitating viral replication, very few cell deaths and/or nuclear changes in neuronal and glial cells were observed at the time when the mice were completely paralyzed. Similar observations were made by Hase, et al. [10] in the study of intracerebral infections of mouse brains by the closely related Japanese encephalitis virus (JEV). They commented that “the JEV does not appear to be strongly cytolytic, and it is conceivable that the cause of death for the mice is severe neuronal dysfunction rather than neuronal destruction”. This was in sharp contrast to our autopsy study in patients who died of severe dengue hemorrhagic fever. Severe liver cell damage was observed in the face of minimal replicative structural changes.

One obvious explanation was the long duration of virus infection in all patients, passing the active period of virus replication known to coincide with the early pyrexic phase of the disease. The ER structural changes might have therefore already been resolved. At the time of autopsy the patients had already been infected for a length of time, which included the symptomatic (4 to 11 days, see Materials and Methods) and the incubation periods. It has been documented that the incubation time of DHF varies widely from 4 to 10 days [11,12]; this means that the patients have been infected for a total length of 8 to 21 days. However, we were surprised by the rapidity of the disappearance and the paucity of DV-induced ER features, and the rare number of virions in the groups of patients who died early (only 4-5 days of illness, Table 1). We would like to speculate that liver injury independent from virus infection (discussed below) might contribute to this acceleration. Without pathology information at the early phase of disease (which is not practical), it is difficult to gauge the contribution of liver to the total pool of virus in patients with different severities. The absence of ultrastructural evidence of virus replication in autopsy material has rendered the technology unsuitable for dengue virus diagnosis, unlike the immunohistochemistry and dengue RNA identification that are more sensitive and suitable.

Similar to this study, a key light microscopic feature of liver pathology reported in our former study, was the prominence of steatosis. Since steatosis has not been a feature shown in the in vitro DV infection of liver cell lines, this pointed to other processes. This is in accord with our previous report that there was no correlation between viral antigen staining (or the quantity of viral RNA found in tissues) and the degree of liver damage, suggesting that DV infection might not be the key contributor [8]. Steatosis was the key pathologic feature in acute alcoholic and other non-alcoholic injuries such as tetracycline toxicity or in Reye’s syndrome [13]. One of the hypotheses on the mechanism of liver damage was that the breakdown of the intestinal barrier allows bacterial pyrogens to reach the liver—a process also known as microbial translocation. Recent findings of raised blood levels of lipopolysaccharide in DHF patients, which correlated with disease severity, offered one possible factor that could injure the liver and suggested that the DV infection of the gut-associated-lymphoid tissues triggered microbial translocation injury to the liver [14]. This speculation remains to be further investigated.

In conclusion, no virus-induced endoplasmic replicating structures have been identified in hepatocytes from DHF patients. Prominent findings were extensive cellular damage and steatosis. The latter process might accelerate the disappearance of the endoplasmic replicating structure occurred in early phase of the disease. It would be interesting to investigate in future study whether the structure could be identified in other organs found infected by the virus [8]. In addition, this ultrastructural study of DV infection has shown for the first time that hallmark cytoplasmic features previously defined in in vitro DV-infected mammalian and insect cell models also occur in the in vivo DV-infected suckling mouse brain. This in vivo model can serve as an important tool for electron microscopists to explore human and experimental animal models to define organ tropism and the viral replication and/or pathological sites responsible for the pathogenesis of DHF and other closely related flaviviruses. 

## Figures and Tables

**Table 1:** Summary of information of seven dengue hemorrhagic fever (DHF) patients from the report [8] whose liver tissues gave adequate ultrastructure information used in this study.

Patient no.	Age Years	Sex	Day of illness *	Hepatic Necrosis/Steatosis ^1^	Liver (cm) ^0^	Lowest Platelets (/μl)	RT-PCR Liver
1	3.5	F	4(2)	II/III	2	18,000	D3
8	3.4	F	3(3)	I/II	4	10,000	D1
9	9	M	3(1)	II/III	2	95,000	D3
10	6	M	7(4)	III/III	3	95,000	D1
11	6	M	4(3)	II/III	(nr) ^†^	12,000	D4
12	9	F	5(2)	I/I	2	75,000	na
13	3	F	3(4)	II/III	4	35,000	D3

* Total days of fever prior to admission and hospital days until death, in parentheses; ^1^ Hepatic necrosis, Steatosis: Grade I = Necrosis area extend 1/3 from central vein to portal triad, Grade II = 2/3, Grade III = Necrosis area extend from central vein to portal triad; ^0^ Liver enlargement measured in centimeter below right costal margin at mid clavicular line; ^†^ nr no record.
